# Crystal structure of 2-benzoyl­amino-*N*′-(4-hy­droxy­benzyl­idene)-3-(thio­phen-2-yl)prop-2-eno­hydrazide

**DOI:** 10.1107/S2056989016010975

**Published:** 2016-07-12

**Authors:** Karanth N. Subbulakshmi, Badiadka Narayana, Hemmige S. Yathirajan, Jerry P. Jasinski, Ravindranath S. Rathore, Christopher Glidewell

**Affiliations:** a Department of Chemistry, Mangalore University, Mangalagangothri 574 199, DK, Mangalore, India; b Department of Studies in Chemistry, University of Mysore, Manasagangotri, Mysuru 570 006, India; c Department of Chemistry, Keene State College, 229 Main Street, Keene, NH 03435-2001, USA; d Department of Bioinformatics, Central University of South Bihar, BIT Campus, PO B. V. College, Patna 800 014, India; e School of Chemistry, University of St Andrews, Fife KY16 9ST, Scotland

**Keywords:** crystal structure, supra­molecular structure, mol­ecular conformation, hydrogen bonding

## Abstract

In the title compound, C_21_H_17_N_3_O_3_S, the non-H atoms, apart from those in the benzoyl group, are almost coplanar (r.m.s. deviation = 0.049 Å) and the benzoyl group is almost orthogonal to the plane of the rest of the mol­ecule [dihedral angle = 80.34 (6)°]. In the crystal, a combination of N—H⋯O and asymmetric bifurcated O—H⋯(N,O) hydrogen bonds link the mol­ecules into a three-dimensional network. Weak C—H⋯O inter­actions are also observed.

## Abstract 2

In the crystal a combination of N—H⋯O and asymmetric bifurcated O—H⋯(N,O) hydrogen bonds link the mol­ecules into a three-dimensional network.

## Chemical context   

Compounds containing hydrazide and Schiff base functionality are of inter­est as examples of this class have been shown to exhibit anti­fungal (Singh & Dash, 1988 ▸), anti-inflammatory (Todeschini *et al.*, 1998 ▸), anti­microbial (Pandeya *et al.*, 1999 ▸) and anti­tumour activity (Desai *et al.*, 2001 ▸).
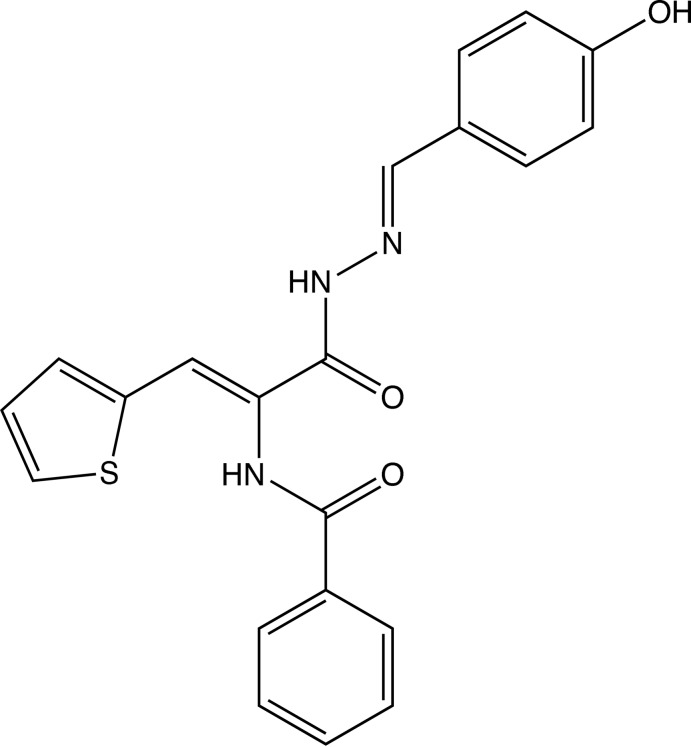


**Figure 1:**
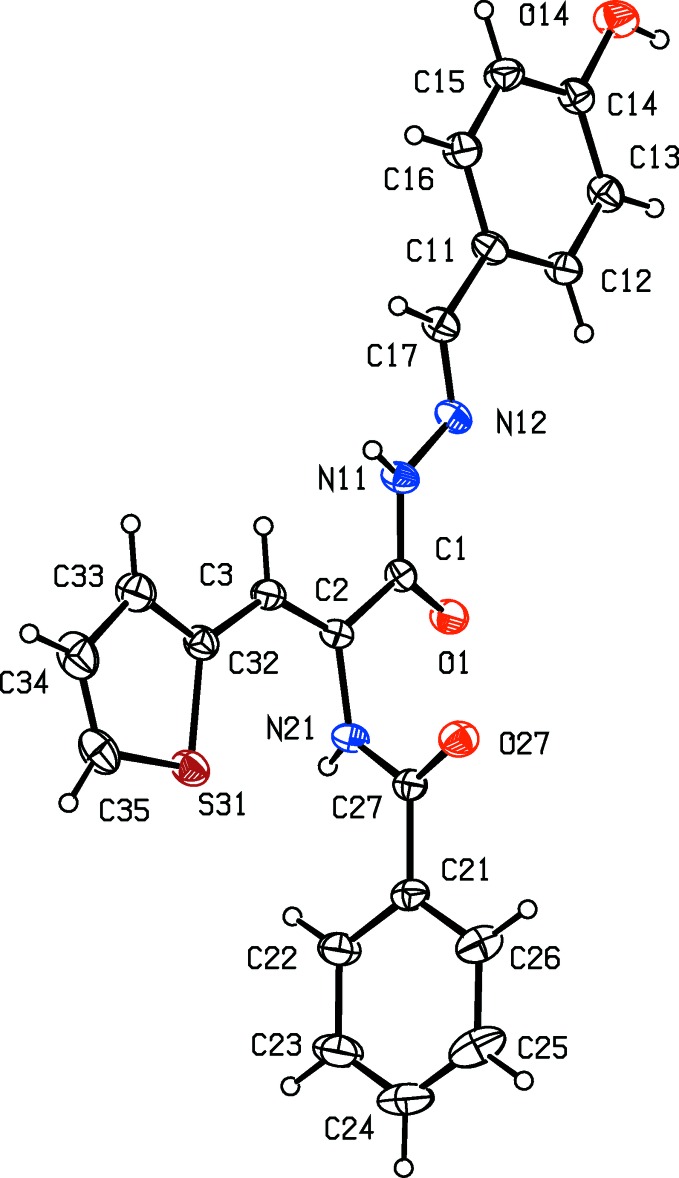
The mol­ecular structure of compound (I), showing displacement ellipsoids drawn at the 30% probability level.

We report here the crystal structure of the title compound, (I) (Fig. 1 ▸), which we compare with the closely related compound methyl 2-benzoyl­amino-3-(thio­phen-2-yl)prop-2-enoate, (II) (Subbulakshmi *et al.*, 2015 ▸). The constitutions of compounds (I) and (II) differ simply in the notional replacement of the COOMe unit in (II) by the CONHN=CHC_6_H_4_OH group in (I). Compound (I) was prepared by condensation of 2-benzoyl­amino-3-(thio­phen-2-yl)prop-2-enoylhydrazine with 4-hy­droxy­benzaldehyde, whereas compound (II) was prepared by the hydrolytic ring-opening of 2-phenyl-4-[(thio­phen-2-yl)-methyl­idene]-1,3-oxazol-5(4*H*)-one to form 2-(benzoyl­amino)-3-(thio­phen-2-yl)prop-2-enoic acid, followed by esterification.

## Structural commentary   

The central core of the mol­ecule of (I), encompassing atoms N21, C3, C2, C1, N11, N12, C17 and C11, is roughly planar: the maximum deviation of any of the component atoms from the mean plane is 0.0859 (14) Å with an r.m.s. deviation of 0.049 Å. The thienyl ring and the aryl ring (C11–C16) are both nearly coplanar with the central spacer unit, making dihedral angles of 1.60 (12) and 5.35 (11)°, respectively. By contrast, the aryl ring (C21–C26) is almost orthogonal to the central unit, making a dihedral angle of 80.34 (6)°. The mol­ecules of (I) exhibit no inter­nal symmetry and they are thus conformationally chiral: the centrosymmetric space group confirms that compound (I) crystallizes as a conformational racemate. The bond distances show clearly that the bonds C2—C3 and N12—C17 are localized double bonds, consistent with the location of the H atoms as deduced from difference maps, ruling out the occurrence in the crystal of any other tautomeric forms. The non-bonded intra­molecular distance O1⋯O27, 3.820 (3) Å, rules out any possibility of an intra­molecular O—H⋯O hydrogen bond.

## Supra­molecular inter­actions   

In the crystal, the mol­ecules of (I) are linked into a three-dimensional network by a combination of two N—H⋯O hydrogen bonds and a three-centre (bifurcated) O—H⋯(N,O) hydrogen bond (Table 1 ▸). The three-centre inter­action is planar within experimental uncertainty with both acceptors in the same mol­ecule, and it is markedly asymmetric. While the longer component might, perhaps, be regarded as an adventitious contact given the proximity of the two acceptor sites, the great propensity of hydroxyl groups to act as hydrogen-bond donors (Desiraju & Steiner, 1999 ▸) cautions against this inter­pretation. Very asymmetric three-centre hydrogen bonds are, in fact, not uncommon: for example, in the structure of 2-amino-4,6-dimeth­oxy-5-nitro­sopyrimidine–water (4/3) (Glidewell *et al.*, 2002 ▸) there are six different three-centre hydrogen bonds, two of which, both of O—H⋯(N,O) type, show asymmetries comparable with that found here in (I); markedly asymmetric N—H⋯(N,O) systems occur in the structures of 2-amino-4,6-bis­(benz­yloxy)-5-nitro­sopyrimidine (Quesada *et al.*, 2002 ▸), and in (*E*)-3-di­methyl­amino-2-(1*H*-indol-3-ylcarbon­yl)acrylo­nitrile, where the two acceptors form parts of different mol­ecules (Galvez *et al.*, 2008 ▸); and a very asymmetric N—H⋯(O)_2_ hydrogen bond having the two acceptors in different mol­ecules occurs in the structure of 3,3-di­fluoro-5-nitro-1*H*-indol-2(3*H*)-one (Glidewell *et al.*, 2005 ▸).

**Figure 2:**
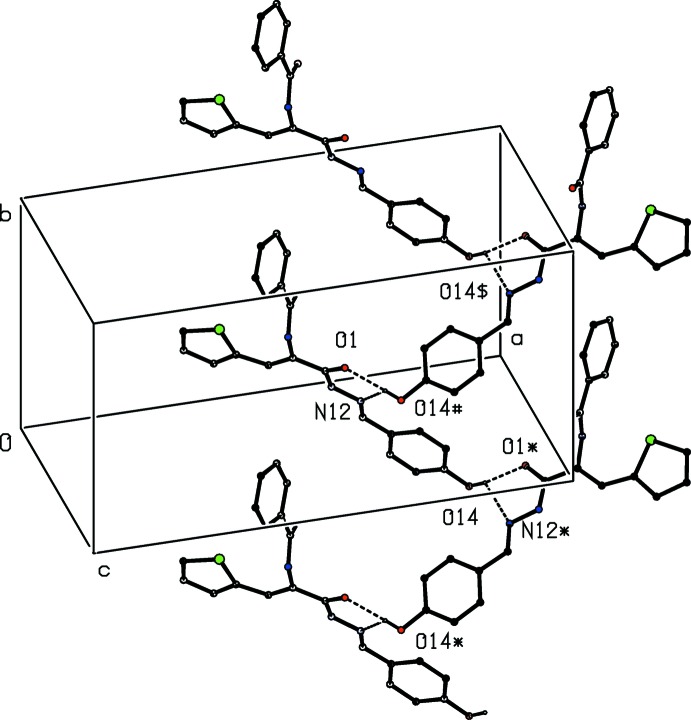
Part of the crystal structure of compound (I), showing the formation of a hydrogen-bonded chain of rings parallel to [010]. Hydrogen bonds are shown as dashed lines and, for the sake of clarity, the H atoms not involved in the motif shown have been omitted. The atoms marked with an asterisk (*), a hash (#) or a dollar sign ($) are at the symmetry positions (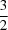 − *x*, −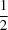 + *y*, 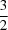 − *z*), (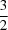 − *x*, 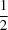 + *y*, 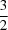 − *z*) and (*x*, 1 + *y*, *z*), respectively.

**Figure 3:**
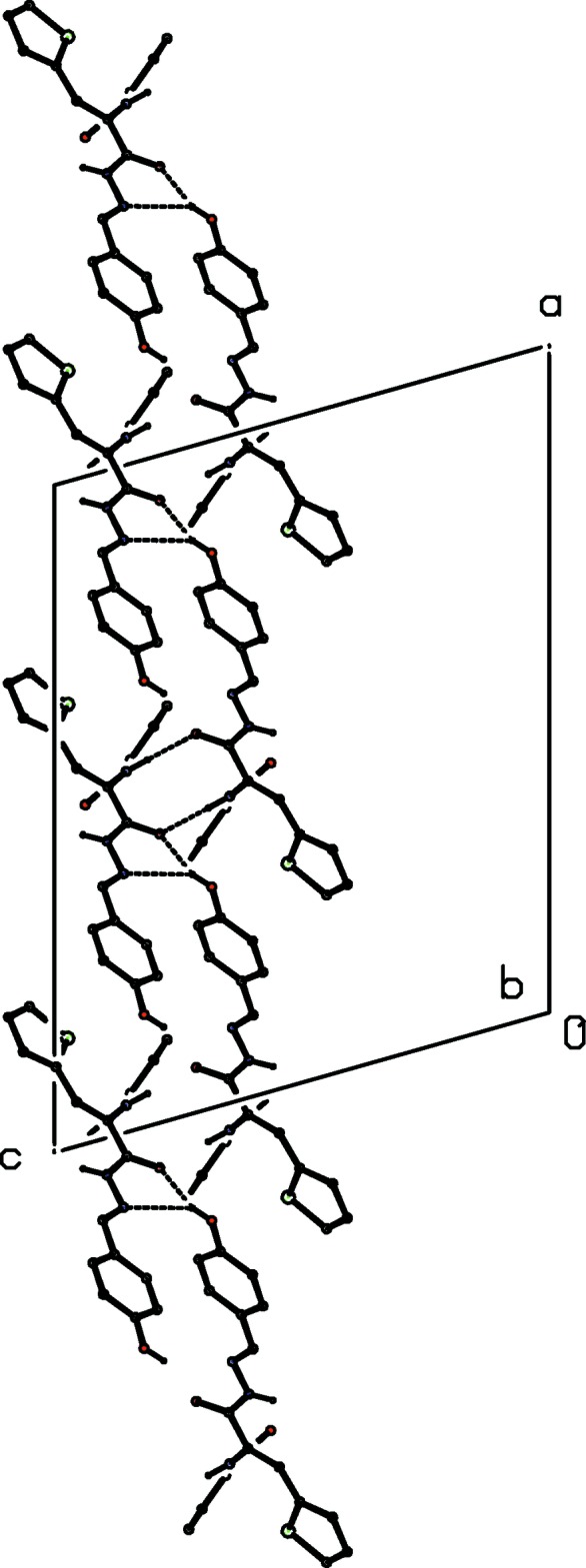
A projection along [010] of part of the crystal structure of compound (I), showing the linking of the [010] chains to form a sheet parallel to (001). Hydrogen bonds are shown as dashed lines and, for the sake of clarity, the H atoms bonded to C atoms have been omitted.

**Figure 4:**
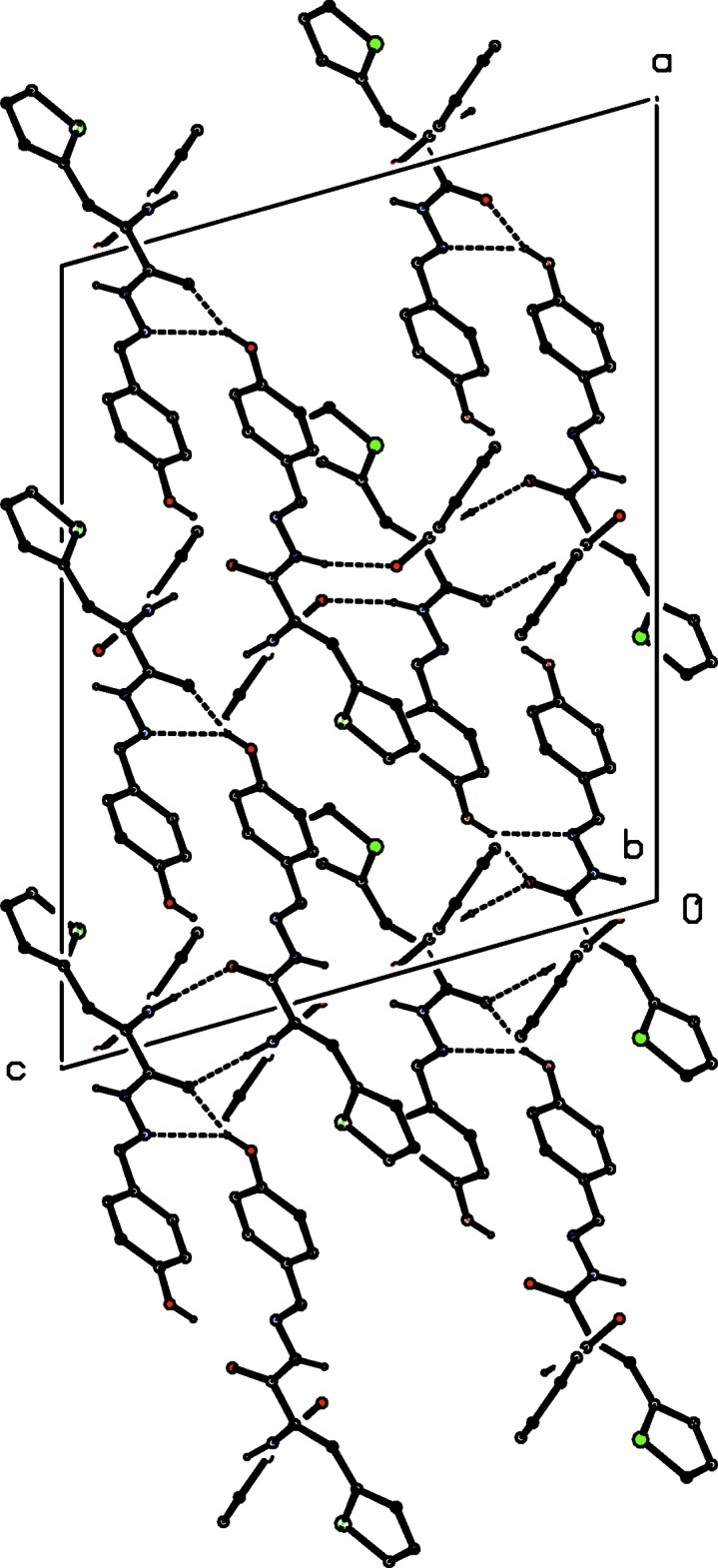
A projection along [010] of part of the crystal structure of compound (I), showing the linking of the (001) sheets to form a three-dimensional framework structure. Hydrogen bonds are shown as dashed lines and, for the sake of clarity, the H atoms bonded to C atoms have been omitted.

The formation of the hydrogen-bonded network in (I) is most readily analysed in terms of simpler substructures of lower dimensionality (Ferguson *et al.*, 1998*a* ▸,*b* ▸; Gregson *et al.*, 2000 ▸). In the simplest of the substructures, mol­ecules related by a 2_1_ screw axis are linked by the three-centre hydrogen bond to form a *C*(8)*C*(11)[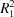
(5)] chain of rings running parallel to the [010] direction (Fig. 2 ▸). The chains of this type are linked by the N—H⋯O hydrogen bond having atom O1 as the acceptor (Table 1 ▸) to form a two-dimensional substructure in the form of a sheet lying parallel to (001) (Fig. 3 ▸). Finally, these sheets are linked by the N—H⋯O hydrogen bond having atom O27 as the acceptor to form a continuous framework structure (Fig. 4 ▸). This network is reinforced by a number of weak C—H⋯O inter­actions (Table 1 ▸), but these are not essential to its formation.

## Database survey   

In the crystal structure of compound (II) (Subbulakshmi *et al.*, 2015 ▸), a combination of N—H⋯O and C—H⋯π(arene) hydrogen bonds links the mol­ecules into sheets; in the structure of (*E*)-*N*′-[1-(2-hy­droxy­phen­yl)ethyl­idene]-3-meth­oxy­benzohydrazide the mol­ecules are linked by a single N—H⋯O hydrogen bond to form simple *C*(4) chains (Li & Ban, 2009 ▸); and the mol­ecules of (*E*)-*N*′-(4-hy­droxy­benzyl­idene)-3-nitro­benzohydrazide are linked into sheets by a combination of N—H⋯O, O—H⋯·(N,O) and C—H⋯O hydrogen bonds (Meng *et al.*, 2012 ▸).

## Synthesis and crystallization   

A mixture of 2-benzoyl­amino-3-(thio­phen-2-yl)prop-2-enoylhydrazine (2.87 g, 0.01 mol), and 4-hy­droxy­benzaldehyde (1.22 g, 0.01 mol) in ethanol (20 ml) was stirred at ambient temperature for 4 h. The resulting solid product was collected by filtration, washed with cold water, dried in air and recrystallized from ethanol solution: m.p. 534–535 K. Crystals of (I) were grown by slow evaporation at room temperature of a solution in 1,4-dioxane-methanol (1:1, *v*/*v*).

## Refinement   

Crystal data, data collection and structure refinement details are summarized in Table 2 ▸. All H atoms were located in difference maps. The H atoms bonded to C atoms were then treated as riding atoms in geometrically idealized positions with C—H = 0.93 Å and with *U*_iso_(H) = 1.2 *U*_eq_(C). For the H atoms bonded to O or N atoms, the atomic coordinates were refined with *U*_iso_(H) = 1.2 *U*_eq_(N) or 1.5*U*_eq_(O), giving the O—H and N—H distances shown in Table 1 ▸.

## Supplementary Material

Crystal structure: contains datablock(s) global, I. DOI: 10.1107/S2056989016010975/hb7597sup1.cif

Structure factors: contains datablock(s) I. DOI: 10.1107/S2056989016010975/hb7597Isup2.hkl

Supporting information file. DOI: 10.1107/S2056989016010975/hb7597Isup3.cml

CCDC reference: 1491115

Additional supporting information: crystallographic information; 3D view; checkCIF report

## Figures and Tables

**Table 1:** Hydrogen-bond geometry (Å, °)

*D*—H⋯*A*	*D*—H	H⋯*A*	*D*⋯*A*	*D*—H⋯*A*
N11—H11⋯O27^i^	0.86 (2)	2.10 (2)	2.9400 (18)	168 (2)
N21—H21⋯O1^ii^	0.827 (19)	2.238 (19)	3.0002 (18)	153.5 (18)
O14—H14⋯O1^iii^	0.84 (3)	1.97 (3)	2.7727 (19)	162 (3)
O14—H14⋯N12^iii^	0.84 (3)	2.59 (3)	3.133 (2)	124 (2)
C3—H3⋯O27^i^	0.93	2.52	3.333 (2)	147
C17—H17⋯O27^i^	0.93	2.57	3.350 (2)	142
C24—H24⋯O14^iv^	0.93	2.58	3.364 (3)	142

Symmetry codes: (i) 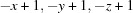; (ii) 
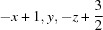
; (iii) 
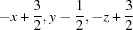
; (iv) 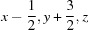.

**Table 2:** Experimental details

Crystal data	
Chemical formula	C_21_H_17_N_3_O_3_S
*M*_r_	391.43
Crystal system, space group	Monoclinic, *C*2/*c*
Temperature (K)	298
*a*, *b*, *c* (Å)	22.5212 (7), 10.1879 (4), 17.3592 (5)
β (°)	105.801 (3)
*V* (Å^3^)	3832.5 (2)
*Z*	8
Radiation type	Mo *K*α
μ (mm^−1^)	0.20
Crystal size (mm)	0.42 × 0.32 × 0.18

Data collection	
Diffractometer	Agilent Xcalibur Eos Gemini
Absorption correction	Multi-scan (*CrysAlis PRO*; Agilent, 2014 ▸)
*T*_min_, *T*_max_	0.757, 0.965
No. of measured, independent and observed [*I* > 2σ(*I*)] reflections	9963, 4419, 3426
*R*_int_	0.024
(sin θ/λ)_max_ (Å^−1^)	0.651

Refinement	
*R*[*F* ^2^ > 2σ(*F* ^2^)], *wR*(*F* ^2^), *S*	0.043, 0.113, 1.04
No. of reflections	4419
No. of parameters	262
H-atom treatment	H atoms treated by a mixture of independent and constrained refinement
Δρ_max_, Δρ_min_ (e Å^−3^)	0.24, −0.26

Computer programs: *CrysAlis PRO* (Agilent, 2014 ▸), *SHELXS97* (Sheldrick, 2008 ▸), *SHELXL2014* (Sheldrick, 2015 ▸) and *PLATON* (Spek, 2009 ▸).

## supplementary crystallographic information

### Crystal data

**Table:**

C_21_H_17_N_3_O_3_S	*F*(000) = 1632
*M**_r_* = 391.43	*D*_x_ = 1.357 Mg m^−^^3^
Monoclinic, *C*2/*c*	Mo *K*α radiation, λ = 0.71073 Å
*a* = 22.5212 (7) Å	Cell parameters from 6327 reflections
*b* = 10.1879 (4) Å	θ = 3.4–32.0°
*c* = 17.3592 (5) Å	µ = 0.20 mm^−^^1^
β = 105.801 (3)°	*T* = 298 K
*V* = 3832.5 (2) Å^3^	Plate, colourless
*Z* = 8	0.42 × 0.32 × 0.18 mm

### Data collection

**Table:**

Agilent Xcalibur Eos Gemini diffractometer	3426 reflections with *I* > 2σ(*I*)
Detector resolution: 16.0416 pixels mm^-1^	*R*_int_ = 0.024
φ and ω scans	θ_max_ = 27.6°, θ_min_ = 3.4°
Absorption correction: multi-scan (CrysAlis PRO; Agilent, 2014)	*h* = −25→29
*T*_min_ = 0.757, *T*_max_ = 0.965	*k* = −13→7
9963 measured reflections	*l* = −20→22
4419 independent reflections	

### Refinement

**Table:**

Refinement on *F*^2^	0 restraints
Least-squares matrix: full	Hydrogen site location: mixed
*R*[*F*^2^ > 2σ(*F*^2^)] = 0.043	H atoms treated by a mixture of independent and constrained refinement
*wR*(*F*^2^) = 0.113	*w* = 1/[σ^2^(*F*_o_^2^) + (0.0475*P*)^2^ + 2.5641*P*] where *P* = (*F*_o_^2^ + 2*F*_c_^2^)/3
*S* = 1.04	(Δ/σ)_max_ = 0.001
4419 reflections	Δρ_max_ = 0.24 e Å^−^^3^
262 parameters	Δρ_min_ = −0.26 e Å^−^^3^

### Special details

**Table:**

Geometry. All esds (except the esd in the dihedral angle between two l.s. planes) are estimated using the full covariance matrix. The cell esds are taken into account individually in the estimation of esds in distances, angles and torsion angles; correlations between esds in cell parameters are only used when they are defined by crystal symmetry. An approximate (isotropic) treatment of cell esds is used for estimating esds involving l.s. planes.

### Fractional atomic coordinates and isotropic or equivalent isotropic displacement parameters (Å^2^)

**Table:**

	*x*	*y*	*z*	*U*_iso_*/*U*_eq_	
C1	0.53659 (7)	0.43211 (16)	0.64694 (9)	0.0306 (3)	
O1	0.56722 (5)	0.47222 (13)	0.71288 (7)	0.0423 (3)	
C2	0.47457 (7)	0.48794 (16)	0.60847 (9)	0.0299 (3)	
C3	0.43369 (7)	0.43455 (17)	0.54526 (9)	0.0347 (4)	
H3	0.4469	0.3586	0.5252	0.042*	
N11	0.55560 (6)	0.33591 (15)	0.60668 (8)	0.0348 (3)	
H11	0.5363 (9)	0.3157 (19)	0.5583 (12)	0.042*	
N12	0.61135 (6)	0.27363 (15)	0.63980 (8)	0.0350 (3)	
C17	0.62189 (8)	0.17766 (18)	0.59827 (10)	0.0372 (4)	
H17	0.5924	0.1570	0.5509	0.045*	
C11	0.67784 (7)	0.09896 (17)	0.62156 (10)	0.0349 (4)	
C12	0.72750 (8)	0.13084 (18)	0.68643 (10)	0.0382 (4)	
H12	0.7261	0.2068	0.7156	0.046*	
C13	0.77876 (8)	0.05097 (19)	0.70785 (10)	0.0393 (4)	
H13	0.8115	0.0730	0.7515	0.047*	
C14	0.78174 (8)	−0.06236 (18)	0.66441 (10)	0.0373 (4)	
O14	0.83122 (6)	−0.14325 (14)	0.68163 (9)	0.0524 (4)	
H14	0.8583 (12)	−0.115 (3)	0.7211 (15)	0.079*	
C15	0.73300 (8)	−0.09439 (18)	0.59955 (11)	0.0436 (4)	
H15	0.7346	−0.1700	0.5701	0.052*	
C16	0.68189 (8)	−0.01392 (19)	0.57854 (10)	0.0421 (4)	
H16	0.6494	−0.0358	0.5345	0.050*	
N21	0.45947 (6)	0.60302 (14)	0.64533 (8)	0.0326 (3)	
H21	0.4510 (9)	0.5932 (19)	0.6884 (11)	0.039*	
C27	0.46800 (7)	0.72320 (17)	0.61800 (9)	0.0314 (3)	
O27	0.49198 (6)	0.73798 (13)	0.56269 (7)	0.0435 (3)	
C21	0.44374 (8)	0.83722 (17)	0.65374 (9)	0.0360 (4)	
C22	0.40355 (9)	0.8231 (2)	0.70116 (12)	0.0497 (5)	
H22	0.3933	0.7395	0.7149	0.060*	
C23	0.37867 (11)	0.9317 (3)	0.72807 (14)	0.0642 (6)	
H23	0.3519	0.9210	0.7600	0.077*	
C24	0.39294 (12)	1.0539 (2)	0.70834 (14)	0.0676 (7)	
H24	0.3756	1.1267	0.7263	0.081*	
C25	0.43304 (14)	1.0707 (2)	0.66171 (15)	0.0737 (7)	
H25	0.4430	1.1548	0.6485	0.088*	
C26	0.45857 (11)	0.9615 (2)	0.63441 (12)	0.0551 (5)	
H26	0.4857	0.9727	0.6030	0.066*	
S31	0.33408 (2)	0.61148 (5)	0.52709 (3)	0.04314 (14)	
C32	0.37235 (8)	0.47716 (18)	0.50374 (10)	0.0367 (4)	
C33	0.33582 (9)	0.4137 (2)	0.43779 (12)	0.0518 (5)	
H33	0.3482	0.3392	0.4153	0.062*	
C34	0.27760 (9)	0.4737 (3)	0.40789 (12)	0.0597 (6)	
H34	0.2473	0.4425	0.3640	0.072*	
C35	0.27068 (9)	0.5808 (2)	0.44974 (12)	0.0533 (5)	
H35	0.2353	0.6323	0.4378	0.064*	

### Atomic displacement parameters (Å^2^)

**Table:**

	*U*^11^	*U*^22^	*U*^33^	*U*^12^	*U*^13^	*U*^23^
C1	0.0294 (8)	0.0327 (8)	0.0303 (7)	0.0004 (6)	0.0090 (6)	0.0012 (6)
O1	0.0340 (6)	0.0506 (8)	0.0378 (6)	0.0050 (5)	0.0019 (5)	−0.0115 (6)
C2	0.0310 (8)	0.0298 (8)	0.0301 (7)	0.0047 (6)	0.0107 (6)	0.0002 (6)
C3	0.0330 (8)	0.0346 (9)	0.0370 (8)	0.0039 (7)	0.0103 (7)	−0.0042 (7)
N11	0.0316 (7)	0.0407 (8)	0.0293 (6)	0.0108 (6)	0.0037 (6)	−0.0008 (6)
N12	0.0307 (7)	0.0406 (8)	0.0335 (7)	0.0096 (6)	0.0086 (6)	0.0043 (6)
C17	0.0346 (8)	0.0429 (10)	0.0323 (8)	0.0077 (7)	0.0062 (7)	0.0036 (7)
C11	0.0338 (8)	0.0383 (9)	0.0330 (8)	0.0069 (7)	0.0097 (7)	0.0043 (7)
C12	0.0378 (9)	0.0399 (10)	0.0368 (8)	0.0055 (7)	0.0103 (7)	−0.0036 (7)
C13	0.0332 (8)	0.0485 (11)	0.0335 (8)	0.0033 (8)	0.0047 (7)	0.0003 (8)
C14	0.0337 (8)	0.0367 (9)	0.0401 (9)	0.0080 (7)	0.0078 (7)	0.0092 (7)
O14	0.0425 (7)	0.0490 (8)	0.0558 (8)	0.0182 (6)	−0.0034 (6)	−0.0021 (7)
C15	0.0417 (10)	0.0354 (10)	0.0488 (10)	0.0076 (8)	0.0043 (8)	−0.0061 (8)
C16	0.0360 (9)	0.0439 (11)	0.0402 (9)	0.0058 (8)	0.0000 (7)	−0.0040 (8)
N21	0.0376 (7)	0.0343 (8)	0.0279 (6)	0.0073 (6)	0.0123 (6)	−0.0011 (6)
C27	0.0296 (8)	0.0349 (9)	0.0266 (7)	0.0033 (6)	0.0023 (6)	−0.0035 (6)
O27	0.0537 (8)	0.0439 (8)	0.0375 (6)	−0.0012 (6)	0.0203 (6)	−0.0028 (5)
C21	0.0363 (9)	0.0357 (9)	0.0305 (8)	0.0061 (7)	−0.0002 (7)	−0.0061 (7)
C22	0.0499 (11)	0.0483 (12)	0.0534 (11)	0.0079 (9)	0.0185 (9)	−0.0102 (9)
C23	0.0613 (14)	0.0656 (16)	0.0688 (14)	0.0177 (12)	0.0231 (11)	−0.0200 (12)
C24	0.0798 (17)	0.0531 (14)	0.0641 (14)	0.0248 (12)	0.0095 (12)	−0.0189 (11)
C25	0.108 (2)	0.0359 (12)	0.0719 (15)	0.0058 (13)	0.0156 (15)	−0.0041 (11)
C26	0.0741 (15)	0.0385 (11)	0.0527 (11)	0.0012 (10)	0.0175 (11)	−0.0040 (9)
S31	0.0342 (2)	0.0489 (3)	0.0449 (3)	0.00813 (19)	0.00828 (18)	−0.0011 (2)
C32	0.0326 (8)	0.0431 (10)	0.0338 (8)	0.0015 (7)	0.0080 (7)	−0.0027 (7)
C33	0.0381 (10)	0.0649 (14)	0.0475 (10)	0.0022 (9)	0.0034 (8)	−0.0158 (9)
C34	0.0363 (10)	0.0921 (18)	0.0430 (10)	0.0006 (11)	−0.0025 (8)	−0.0101 (11)
C35	0.0328 (9)	0.0762 (15)	0.0471 (11)	0.0103 (9)	0.0047 (8)	0.0074 (10)

### Geometric parameters (Å, º)

**Table:**

C1—O1	1.2342 (19)	N21—C27	1.346 (2)
C1—N11	1.340 (2)	N21—H21	0.826 (19)
C1—C2	1.487 (2)	C27—O27	1.2322 (19)
C2—C3	1.341 (2)	C27—C21	1.489 (2)
C2—N21	1.421 (2)	C21—C26	1.374 (3)
C3—C32	1.440 (2)	C21—C22	1.387 (3)
C3—H3	0.9300	C22—C23	1.378 (3)
N11—N12	1.3844 (18)	C22—H22	0.9300
N11—H11	0.859 (19)	C23—C24	1.353 (4)
N12—C17	1.275 (2)	C23—H23	0.9300
C17—C11	1.455 (2)	C24—C25	1.378 (4)
C17—H17	0.9300	C24—H24	0.9300
C11—C16	1.388 (2)	C25—C26	1.394 (3)
C11—C12	1.392 (2)	C25—H25	0.9300
C12—C13	1.378 (2)	C26—H26	0.9300
C12—H12	0.9300	S31—C35	1.702 (2)
C13—C14	1.391 (3)	S31—C32	1.7235 (18)
C13—H13	0.9300	C32—C33	1.376 (2)
C14—O14	1.352 (2)	C33—C34	1.411 (3)
C14—C15	1.381 (2)	C33—H33	0.9300
O14—H14	0.84 (3)	C34—C35	1.343 (3)
C15—C16	1.379 (2)	C34—H34	0.9300
C15—H15	0.9300	C35—H35	0.9300
C16—H16	0.9300		
			
O1—C1—N11	123.27 (15)	C27—N21—H21	121.3 (14)
O1—C1—C2	120.61 (15)	C2—N21—H21	116.8 (14)
N11—C1—C2	116.11 (13)	O27—C27—N21	121.39 (15)
C3—C2—N21	120.54 (14)	O27—C27—C21	121.18 (16)
C3—C2—C1	124.32 (15)	N21—C27—C21	117.32 (14)
N21—C2—C1	115.10 (13)	C26—C21—C22	118.82 (18)
C2—C3—C32	129.69 (16)	C26—C21—C27	118.41 (17)
C2—C3—H3	115.2	C22—C21—C27	122.62 (17)
C32—C3—H3	115.2	C23—C22—C21	120.6 (2)
C1—N11—N12	120.08 (13)	C23—C22—H22	119.7
C1—N11—H11	122.5 (13)	C21—C22—H22	119.7
N12—N11—H11	117.2 (13)	C24—C23—C22	120.5 (2)
C17—N12—N11	113.83 (14)	C24—C23—H23	119.8
N12—C17—C11	123.09 (15)	C22—C23—H23	119.8
N12—C17—H17	118.5	C23—C24—C25	120.1 (2)
C11—C17—H17	118.5	C23—C24—H24	119.9
C16—C11—C12	118.20 (15)	C25—C24—H24	119.9
C16—C11—C17	119.08 (15)	C24—C25—C26	119.9 (2)
C12—C11—C17	122.71 (16)	C24—C25—H25	120.1
C13—C12—C11	120.66 (17)	C26—C25—H25	120.1
C13—C12—H12	119.7	C21—C26—C25	120.2 (2)
C11—C12—H12	119.7	C21—C26—H26	119.9
C12—C13—C14	120.29 (16)	C25—C26—H26	119.9
C12—C13—H13	119.9	C35—S31—C32	91.97 (10)
C14—C13—H13	119.9	C33—C32—C3	123.35 (17)
O14—C14—C15	117.42 (17)	C33—C32—S31	110.17 (14)
O14—C14—C13	123.00 (16)	C3—C32—S31	126.48 (13)
C15—C14—C13	119.57 (15)	C32—C33—C34	112.82 (19)
C14—O14—H14	110.1 (19)	C32—C33—H33	123.6
C16—C15—C14	119.75 (17)	C34—C33—H33	123.6
C16—C15—H15	120.1	C35—C34—C33	112.70 (18)
C14—C15—H15	120.1	C35—C34—H34	123.7
C15—C16—C11	121.52 (16)	C33—C34—H34	123.7
C15—C16—H16	119.2	C34—C35—S31	112.35 (15)
C11—C16—H16	119.2	C34—C35—H35	123.8
C27—N21—C2	121.16 (13)	S31—C35—H35	123.8
			
O1—C1—C2—C3	−166.88 (16)	C2—N21—C27—O27	−3.6 (2)
N11—C1—C2—C3	11.8 (2)	C2—N21—C27—C21	172.62 (13)
O1—C1—C2—N21	11.0 (2)	O27—C27—C21—C26	−11.9 (2)
N11—C1—C2—N21	−170.33 (14)	N21—C27—C21—C26	171.89 (16)
N21—C2—C3—C32	1.0 (3)	O27—C27—C21—C22	163.63 (16)
C1—C2—C3—C32	178.77 (16)	N21—C27—C21—C22	−12.6 (2)
O1—C1—N11—N12	1.9 (3)	C26—C21—C22—C23	0.3 (3)
C2—C1—N11—N12	−176.74 (14)	C27—C21—C22—C23	−175.14 (17)
C1—N11—N12—C17	175.03 (16)	C21—C22—C23—C24	0.3 (3)
N11—N12—C17—C11	179.60 (16)	C22—C23—C24—C25	−0.6 (4)
N12—C17—C11—C16	171.54 (17)	C23—C24—C25—C26	0.4 (4)
N12—C17—C11—C12	−7.3 (3)	C22—C21—C26—C25	−0.5 (3)
C16—C11—C12—C13	−1.0 (3)	C27—C21—C26—C25	175.14 (18)
C17—C11—C12—C13	177.86 (16)	C24—C25—C26—C21	0.1 (4)
C11—C12—C13—C14	0.5 (3)	C2—C3—C32—C33	178.32 (19)
C12—C13—C14—O14	178.76 (17)	C2—C3—C32—S31	−1.3 (3)
C12—C13—C14—C15	0.1 (3)	C35—S31—C32—C33	0.22 (16)
O14—C14—C15—C16	−178.83 (17)	C35—S31—C32—C3	179.89 (17)
C13—C14—C15—C16	−0.1 (3)	C3—C32—C33—C34	179.77 (18)
C14—C15—C16—C11	−0.5 (3)	S31—C32—C33—C34	−0.6 (2)
C12—C11—C16—C15	1.0 (3)	C32—C33—C34—C35	0.7 (3)
C17—C11—C16—C15	−177.89 (18)	C33—C34—C35—S31	−0.5 (3)
C3—C2—N21—C27	−86.1 (2)	C32—S31—C35—C34	0.18 (18)
C1—C2—N21—C27	95.92 (17)		

### Hydrogen-bond geometry (Å, º)

**Table:**

*D*—H···*A*	*D*—H	H···*A*	*D*···*A*	*D*—H···*A*
N11—H11···O27^i^	0.86 (2)	2.10 (2)	2.9400 (18)	168 (2)
N21—H21···O1^ii^	0.827 (19)	2.238 (19)	3.0002 (18)	153.5 (18)
O14—H14···O1^iii^	0.84 (3)	1.97 (3)	2.7727 (19)	162 (3)
O14—H14···N12^iii^	0.84 (3)	2.59 (3)	3.133 (2)	124 (2)
C3—H3···O27^i^	0.93	2.52	3.333 (2)	147
C17—H17···O27^i^	0.93	2.57	3.350 (2)	142
C24—H24···O14^iv^	0.93	2.58	3.364 (3)	142

Symmetry codes: (i) −*x*+1, −*y*+1, −*z*+1; (ii) −*x*+1, *y*, −*z*+3/2; (iii) −*x*+3/2, *y*−1/2, −*z*+3/2; (iv) *x*−1/2, *y*+3/2, *z*.
